# Achieving change in primary care—effectiveness of strategies for improving implementation of complex interventions: systematic review of reviews

**DOI:** 10.1136/bmjopen-2015-009993

**Published:** 2015-12-22

**Authors:** Rosa Lau, Fiona Stevenson, Bie Nio Ong, Krysia Dziedzic, Shaun Treweek, Sandra Eldridge, Hazel Everitt, Anne Kennedy, Nadeem Qureshi, Anne Rogers, Richard Peacock, Elizabeth Murray

**Affiliations:** 1 eHealth Unit, Department of Primary Care and Population Health, University College London, London, UK; 2 Arthritis Research UK Primary Care Centre, Research Institute for Primary Care Sciences and Health Sciences, Keele University, Keele, UK; 3 Health Services Research Unit, University of Aberdeen, Scotland, UK; 4 Centre for Primary Care and Public Health, Queen Mary University of London, London, UK; 5 Primary Care and Population Sciences, Faculty of Medicine, University of Southampton, Southampton,UK; 6 Faculty of Health Sciences, NIHR CLAHRC Wessex, University of Southampton, Southampton, UK; 7 Division of Primary Care, University of Nottingham, Derby, UK; 8 Archway Healthcare Library, London, UK

**Keywords:** PRIMARY CARE, Implementation, Systematic review

## Abstract

**Objective:**

To identify, summarise and synthesise available literature on the effectiveness of implementation strategies for optimising implementation of complex interventions in primary care.

**Design:**

Systematic review of reviews.

**Data sources:**

MEDLINE, EMBASE, CINAHL, Cochrane Library and PsychINFO were searched, from first publication until December 2013; the bibliographies of relevant articles were screened for additional reports.

**Eligibility criteria for selecting studies:**

Eligible reviews had to (1) examine effectiveness of single or multifaceted implementation strategies, (2) measure health professional practice or process outcomes and (3) include studies from predominantly primary care in developed countries. Two reviewers independently screened titles/abstracts and full-text articles of potentially eligible reviews for inclusion.

**Data synthesis:**

Extracted data were synthesised using a narrative approach.

**Results:**

91 reviews were included. The most commonly evaluated strategies were those targeted at the level of individual professionals, rather than those targeting organisations or context. These strategies (eg, audit and feedback, educational meetings, educational outreach, reminders) on their own demonstrated a small to modest improvement (2–9%) in professional practice or behaviour with considerable variability in the observed effects. The effects of multifaceted strategies targeted at professionals were mixed and not necessarily more effective than single strategies alone. There was relatively little review evidence on implementation strategies at the levels of organisation and wider context. Evidence on cost-effectiveness was limited and data on costs of different strategies were scarce and/or of low quality.

**Conclusions:**

There is a substantial literature on implementation strategies aimed at changing professional practices or behaviour. It remains unclear which implementation strategies are more likely to be effective than others and under what conditions. Future research should focus on identifying and assessing the effectiveness of strategies targeted at the wider context and organisational levels and examining the costs and cost-effectiveness of implementation strategies.

**PROSPERO registration number:**

CRD42014009410.

**Strengths and limitations of this study**

- To the best of our knowledge, this is the most comprehensive systematic review of reviews to examine the evidence on the effectiveness of single or multifaceted strategies for improving implementation of any kinds of complex interventions in primary care. As a result, 91 relevant reviews were included.
- The review addressed a number of questions and was conducted using rigorous and transparent multistep reviewing methods.
- The review reveals most of the existing research focused on strategies that addressed individual-level barriers. Most of these professional-level strategies were associated with small to modest improvement in professional practice and process outcomes. There is a lack of research on organisational-level strategies and context-level strategies.
- It is possible that not all relevant primary research studies were captured by included reviews (especially those published recently), so some findings may be missed by concentrating on reviews.

## Introduction

Internationally the pace of change in healthcare continues to be rapid with a drive to implement more clinically and cost-effective interventions to improve care. The need to reduce the delay in translating evidence-based interventions into every day clinical practice, known as the ‘second translational gap’, is widely acknowledged.^1^ ^2^ Almost all changes to practice in primary care involve ‘complex interventions’, that is, interventions with multiple interacting components. They include changes in individual clinicians’ diagnostic and treatment approaches, in operational systems including information technology, altered divisions of labour between healthcare professionals and organisation of care; and require change at multiple levels.^3^ For instance, improving hand hygiene practices may appear simple but on closer inspection, it requires change at multiple levels.^4^ At the individual level, education might change attitudes and acceptance; local opinion leaders might be effective motivators; and reminders could prompt hand hygiene. Organisational structure and culture are important to facilitate change in hand hygiene practices including having adequate resources and infrastructure, for example, changing sink location to improve accessibility and convenience, having a continuous supply of hand wash and paper towels.^4^ It is also widely recognised that the policy context, professional and organisational context and political economic circumstances impacting on the healthcare environment impact on the design and implementation of complex interventions.^5^

Ninety per cent of patient contacts take place in primary care in England. Primary care clinicians are generalists and often manage a set of undifferentiated symptoms or health problems; this requires a combination of wide ranging knowledge, clinical experience and sound judgement.^6^ Their roles and activities have changed and expanded over the recent years; for example, they are increasingly likely to be involved in care coordination for people with complex problems and areas of ‘specialist’ care (eg, diagnostics and minor surgery), as a result of the development of new medical technologies.^7^ Furthermore, these clinicians often work as part of a multiprofessional team. In England, primary care has been subject to particularly rapid change since the introduction of the Health and Social Care Act of 2012. All of these make this setting particularly challenging to implement ‘complex interventions’.

Implementation strategies can be defined as techniques or methods aimed at improving or optimising the uptake and implementation of complex interventions into routine care.^8^ In this paper, we use this definition of implementation strategies, and use the term ‘strategy’ where we focus on implementation, to differentiate from the term ‘intervention’ which we use for the clinical intervention being implemented. The Cochrane Effective Practice and Organisation of Care (EPOC) group has developed the EPOC taxonomy of interventions designed to improve the delivery, practice and organisation of healthcare services. This taxonomy divides implementation strategies into (1) professional interventions (strategies targeted at professionals), such as printed educational materials, audit and feedback, educational meetings, computerised and non-computerised reminders, educational outreach visits, local opinion leaders; (2) organisational interventions (strategies targeted at the organisation), such as introducing a new role or way of working; (3) financial interventions (strategies targeted at the wider context) such as incentives or changes in reimbursement structure/method; and (4) regulatory interventions (strategies targeted at the wider context) such as introduction of or change in policy or legislation (see online supplementary file 2).^9^ Strategies may be used alone or in combination and as described in the EPOC taxonomy, may target health professionals, organisations or wider contextual issues.

A systematic review of reviews was deemed to be the appropriate method to address this complex issue as the literature is substantial and heterogeneous, covering different clinical interventions, populations, clinical domains and outcomes. Existing reviews tend to focus either on a particular type of complex intervention (eg, introduction of new technologies or promoting uptake and use of guidelines) or on a particular health condition (eg, mental health or diabetes). No single review provides researchers, managers, clinicians or policymakers with coherent guidance to which strategies are effective at implementing change in primary care.

We aimed to identify, summarise and synthesise the available review literature on the effectiveness of implementation strategies for improving uptake of complex interventions in primary care. This review addressed the following questions:

1. What is the effectiveness of single strategies alone in improving uptake of complex interventions in primary care compared with no strategy or alternative single strategy?
2. What is the effectiveness of (particular combinations of) multifaceted strategies in improving uptake of complex interventions in primary care, compared with no strategy, alternative single strategy or other combinations?
3. Are multifaceted strategies more effective than single strategies (or vice versa)?
4. What are the active components of strategies which appear to be associated with success?
5. What is the cost-effectiveness of available implementation strategies?

## Methods

### Search strategy

A comprehensive electronic search was performed in five databases: MEDLINE, EMBASE, Cumulative Index of Nursing and Allied Health (CINAHL), the Cochrane Library and PsycINFO. The search was performed by the primary reviewer (RL), supported by a specialist librarian (RP). The search strategy was developed using both medical subject headings, for example, ‘translational medical research’, ‘evidence-based practice’, ‘general practice’, ‘review’, ‘review literature as topic’ and free-text words, for example, evidence to practice, evidence practice gap, family doctor, implementation, adoption. Articles reported in English and published up to December 2013 were eligible for inclusion in this review. Citation searches were carried out in ISI Web of Science and reference lists of all included articles were screened for additional literature. Details of the search strategy for MEDLINE are provided in online supplementary file 3.

## Eligibility criteria

Eligibility criteria were defined to enable transparent and reproducible selection of papers for inclusion, using the PICO framework.

*Population*: reviews where at least 50% original studies came from primary care in developed countries.

The Royal College of General Practitioners (RCGP) has defined primary care as “the first level contact with people taking action to improve health in a community”.^10^ Primary care teams are defined as teams or groups of health professionals that include a primary care physician (ie, general practitioners, family physicians and other generalist physicians working in primary care settings). Reviews exclusively on secondary care, dental practices, pharmacies or developing countries were excluded.

*Intervention*: use of single or multifaceted strategies to improve implementation of complex interventions that focus on changing clinical practice (see online supplementary file 2). Studies that aimed to evaluate the efficacy or effectiveness of new models of care (eg, collaborative care model for depression care, case management or other integrated care services) were excluded. In addition, we decided to exclude reviews of clinical decision support systems because the focus of reviews in this area was improvement of clinical outcomes, rather than uptake or use. As this review focused on implementation with the aim of improving healthcare delivery and/or clinical practice, we excluded strategies aimed at directly changing patients’ behaviour.

*Comparator*: usual care, no strategy or a different implementation strategy (either single or multifaceted).

*Outcome*: degree of implementation measures, such as composite professional outcome (eg, adherence to desired practice), measures of process of care (eg, referral rates) and professionals’ performance (eg, prescribing, adherence to guidelines). Papers that reported outcomes related to patient health status or change in professionals’ knowledge (without any reference to behaviour or performance in practice) only were excluded.

*Study types*: systematic reviews (structured search of bibliographic and other databases to identify relevant literature; use of transparent methodological criteria; presentation of rigorous conclusions about outcomes), meta-analyses and narrative reviews (purposive sampling of the literature use of theoretical or topical criteria to include papers on the basis of type, relevance and perceived significance, with the aim of summarising, discussing and critiquing conclusions).^11^ These reviews were carried out by including quantitative primary studies (eg, randomised controlled trials (RCTs), controlled before and after studies) and they are the appropriate study design to investigate the effectiveness of implementation strategies. Original research studies, meta-syntheses of qualitative research papers, secondary analysis of original data (eg, individual patient data meta-analysis), conference abstracts, editorials and commentary articles were excluded.

## Study selection

Duplicate references were deleted. The titles and abstracts of all the records obtained from the search were independently double-screened. The primary review author (RL) screened all identified citations (titles and abstracts) for potential inclusion; co-authors acted as the second reviewers. RL obtained the full text of potentially eligible articles which were assessed for eligibility against the prespecified inclusion and exclusion criteria by two reviewers (RL and EM). Any discordance or uncertainty was resolved through discussion between the two reviewers initially and the involvement of a third reviewer as necessary.

## Data management and extraction

For all eligible full-text articles, data were extracted by a single reviewer (RL) using standardised structured data abstraction forms. The content of the data abstraction forms were reviewed for validity by the co-authors, who have extensive experience in systematic review methodologies and implementation/evaluation of complex interventions, to ensure all key important information from the included reviews were captured. Information about the reviews, including title, aims and objectives, setting, review methodology, number of included primary studies, details of analysis, critical appraisal of included primary studies such as the use of any quality assessment tool, and outcome measures were extracted.

Owing to the substantial literature relevant to this review, a systematic, transparent and rigorous method was developed and applied, to enable more effective and efficient data management and synthesis. In brief, this method involved the following steps: (1) sorting papers according to the EPOC taxonomy; (2) selection of a benchmark review paper for each category; (3) selection of important outcomes; (4) data extraction. Selection of a benchmark review was based on predetermined criteria, namely: rigour of reviewing methodology (quality associated with methods and analysis undertaken), comprehensiveness (scope and breadth of topic) and year of publication (most recent review usually included the highest number of relevant studies). These criteria were developed by all co-authors through consensus, and then applied by one author (RL) and checked by two other authors independently. For example, Forsetlund *et al*^12^ was chosen as the benchmark review paper for continuing medical education because (1) it included the largest number of primary studies covering a number of broad topics, that is, general management of various health conditions such as prescribing behaviour, preventive care, screening; (2) quality appraisal was conducted using appropriate checklists; (3) adjusted median risk difference (RD) and relative percentage change were calculated; and (4) the analysis included only primary studies that were of low/moderate risk of bias. We identified six subsequent reviews that were found to be relevant to continuing medical education, all of which conducted narrative synthesis and did not assess the quality of the included primary studies; one had a relatively limited scope of only focusing on older patients.

As many benchmark reviews reported large numbers of outcomes of varying relevance, we decided to select at least one and no more than three outcomes based on their generalisability, validity and reliability. We operationalised generalisability as the degree to which a given outcome was likely to apply across different settings, validity as the extent to which the measure accurately reflected a desired outcome (eg, a change in prescribing behaviour was prioritised over a change in knowledge), and reliability as the degree to which the measure was likely to give similar results if repeated under similar circumstances. As many of these judgements were subjective, we aimed to achieve consensus among co-authors using the following process: RL extracted all the outcomes from each benchmark review and circulated them to all co-authors, who applied the above criteria to rank the available outcomes. Where there was disagreement between co-authors, further discussion was held until consensus was reached.

Finally, data were fully extracted from each selected benchmark, including characteristics of the review (eg, aim/objectives, databases searched, topic/targeted behaviour, selection criteria, outcome measures) and selected outcomes. Data for both dichotomous and continuous outcome measures were extracted. For dichotomous outcomes, the adjusted RD was usually calculated and reported in the reviews. The RD is the difference in outcome between intervention and control group means postintervention minus the difference between groups before the intervention. For continuous outcomes, the percentage change relative to the control mean postintervention was usually calculated. This is the adjusted difference between the intervention and control group means divided by the postintervention control group mean×100%. Median RD or change relative to the control was preferred as the summary estimate is less likely to be driven by possible outlying results (such as large effects from small studies of poor methodological quality). The interquartile ranges (IQRs), as a measure of the spread of the data, were also extracted. The results of the remaining relevant reviews in each EPOC category were summarised and entered into the synthesis table. Some papers conducted subgroup analyses and metaregression on various predetermined features, most commonly level of complexity (low vs high), type of targeted behaviour, format, and presence or absence of tailoring. This information was extracted if provided, in order to explore potential features associated with implementation success.

### Data synthesis

A narrative approach was employed to synthesise the results of the included reviews using a synthesis table that was structured in accordance with our research questions. The synthesis table allowed comparison of results between benchmark paper and non-benchmark papers for each strategy. An example of this can be found in online supplementary file 5. Results of each non-benchmark paper were summarised (along with effect size if provided) and compared with the results of the benchmark paper. The results were arranged by topic or targeted behaviour ((1) any targeted behaviour; (2) guideline implementation (eg, guideline on asthma, cardiovascular disease); (3) disease management/diagnosis (eg, diabetes, hypertension, dementia); (4) prevention and screening (eg, cervical cancer, breast cancer); (5) prescribing behaviour (eg, antibiotic prescribing for respiratory conditions). Information such as the number or type of included studies and whether quality appraisal of studies was performed, were extracted to help explain potential differences (if applicable) in results between the benchmark and non-benchmark paper. Furthermore, a table (table 2) was developed to record the active components of strategies which appear to be associated with success.

In addition to reporting the size of effect, to aid interpretation, we categorised the results using the definitions proposed by Grimshaw *et al*^13^ for dichotomous outcomes (absolute difference):

- ‘Small’ to describe effect sizes ≤5%;
- ‘Modest’ to describe effect sizes >5% and ≤10%;
- ‘Moderate’ to describe effect sizes >10% and ≤20%;
- ‘Large’ to describe effect sizes >20%.

A flow diagram summarising the steps used to undertake this review of review can be found in online supplementary file 4.

## Quality assessment

A subset of data extraction and synthesis (all benchmark review papers plus two randomly selected subsequent papers for each category) were checked by the co-investigators, using a quality assurance form. The PRISMA (Preferred Reporting Items for Systematic Reviews and Meta-analyses) checklist was used to critically appraise the quality of reporting of the included benchmark review papers. PRISMA is a 27-item checklist consisting of preferred reporting items for systematic reviews and meta-analyses and it is primarily focused on randomised trials and quantitative data.^14^

We reported our findings in accordance with the PRISMA guidelines. The full version of the review protocol was published elsewhere.^15^ This systematic review was part of a National Institute of Health Research (NIHR) School for Primary Care Research (SPCR) funded project (SPCR FR4 project number: 122). The systematic review protocol was registered on the PROSPERO database (CRD42014009410).

## Results

### Identification of relevant reviews

**Figure 1:**
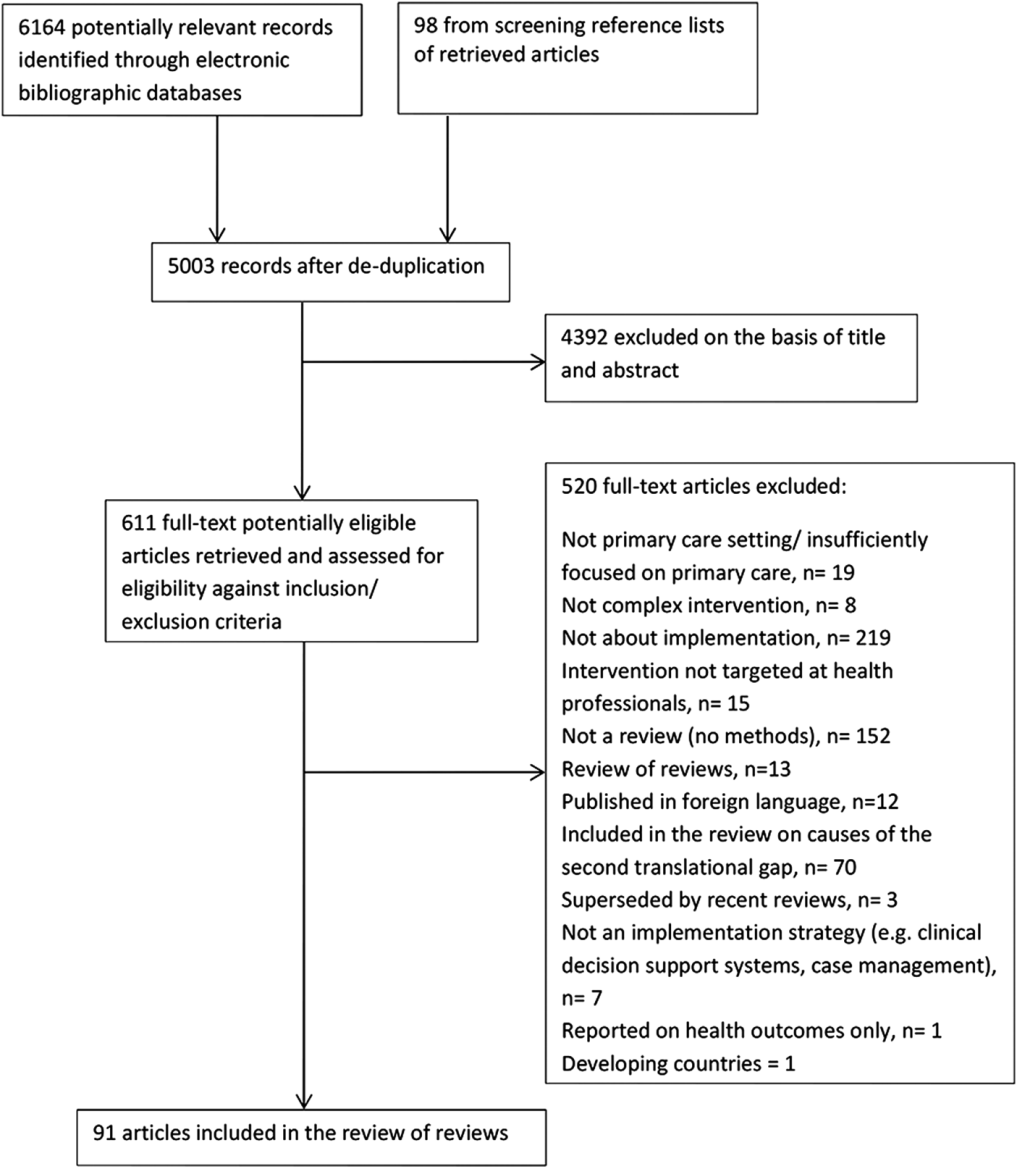
PRISMA flow diagram of study selection.

Searches of the five electronic databases to December 2013 yielded a total of 6164 potentially eligible papers. Following the screening of titles and abstracts and full-text papers, 91 papers were included in the final systematic review of reviews, of which 9 were selected as benchmark reviews. Figure 1 presents the PRISMA flow diagram of study selection.

### Characteristics of included reviews

Details of included reviews are presented in online supplementary table S1. The majority of the included reviews (n=64; 70%) reported data on strategies targeted at individual healthcare professionals (ie, professional-level strategies); with 20 reviews (22%) reporting data on audit and feedback,^13^ ^16–34^ 18 (20%) on printed educational materials,^13^ ^21–24^ ^26^ ^27^ ^32^ ^35–44^ 16 (18%) on educational outreach visits,^13^ ^21^ ^22^ ^26–28^ ^32^ ^35^ ^37^ ^40^ ^45–50^ 26 (29%) on educational meetings,^12^ ^19^ ^21^ ^22^ ^25^ ^31^ ^32^ ^35^ ^38^ ^41^ ^42^ ^47^ ^51–64^ 7 (8%) on local opinion leaders^21^ ^22^ ^24^ ^32^ ^37^ ^40^ ^65^ and 24 (26%) on physician-based reminders.^13^ ^19^ ^21^ ^22^ ^25^ ^28^ ^29^ ^37^ ^40^ ^49^ ^58^ ^60^ ^66–77^ Ten reviews (11%) reported data on organisational implementation strategies (including revising professional roles and facilitation).^28^ ^61^ ^69^ ^78–84^ Eleven reviews (12%) reported data on strategies targeted at the context level; all focused on financial strategies (eg, performance-based payment, fixed fee per patient achieving a specified outcome, single threshold target payment, capitation)^20^ ^23^ ^78^ ^80^ ^85–91^ and we could not identify any reviews on the effectiveness of regulatory strategies. Limited evidence was found on the cost-effectiveness of implementation strategies (economic evaluations, eg, cost-effectiveness, costs benefit analyses were rare).

The focus of included reviews varied: some focused on a specific strategy (eg, audit and feedback) across multiple topic areas and outcomes; others considered the effectiveness of *any* or multiple strategies to improve a *particular* targeted behaviour (eg, cancer screening, guideline adherence); and yet others considered the effectiveness of a specific strategy to improve a particular targeted behaviour (single strategy, single topic area). Seventeen reviews focused on guideline implementation, 13 on quality of care or disease management, 1 on technology implementation, 18 on preventative care, 2 on collaborative working and 4 on prescribing behaviour.

Fifty reviews (71%) were based exclusively in primary care and the remaining in mixed healthcare settings. Twenty-four reviews (26%) were undertaken in the USA, 12 (13%) in Canada, 17 (19%) in the UK, 6 (7%) in Australia, 14 (15%) in Europe and 9 (10%) elsewhere. The original studies included in the reviews were conducted worldwide, although 21 (23%) reported that the original studies were predominantly conducted in the USA. The number of original studies included in the reviews ranged from 2 to 235.

### Methodological quality of included reviews

#### Benchmark reviews

All nine benchmark reviews^12^ ^34^ ^44^ ^50^ ^65^ ^76^ ^82^ ^91^ ^92^ applied a priori criteria for selecting eligible papers and critically appraised the quality of the included primary studies. Five included RCTs only,^12^ ^34^ ^44^ ^50^ ^92^ and four excluded studies that were graded as high risk of bias, or judged to be of poor quality.^12^ ^34^ ^50^ ^82^ Some benchmark reviews used criteria to select the outcomes reported. Where the primary papers described a primary outcome, this was used; where there were multiple outcomes with no named primary outcome, the median value across multiple outcomes was calculated.^12^ ^34^ ^50^ ^65^ All outcomes were expressed as compliance with desired practice (composite outcome) which may include outcomes such as adherence to guidelines, screening rates and appropriate referrals, or process improvements. Eight reviews conducted some form of quantitative analysis (eg, meta-analysis, calculations of median RD, metaregression)^12^ ^34^ ^44^ ^50^ ^65^ ^76^ ^82^ ^92^ and one conducted narrative synthesis.^91^ Quality assessment of all benchmark papers can be found in online supplementary file 6.

#### Other (non-benchmark) reviews

Overall, 79 reviews (96%) reported the use of explicit inclusion/exclusion criteria. Sixteen reviews (20%) included only randomised trials, 59 (72%) included studies with both randomised and non-randomised designs (eg, quasi-experimental, controlled before-after studies, interrupted time series). Eighteen (22%) conducted some form of quantitative analysis (eg, meta-analysis, calculations of median RD, metaregression) and the rest conducted narrative synthesis. Forty-seven reviews (57%) critically appraised their included primary studies using some form of checklist/assessment or described quality issues in the results or discussion. Only one review synthesised data using a theoretical framework.^16^

#### Effects of single strategies

##### Strategies directed at individual professionals

###### Single strategy alone versus no strategy or usual care

**Figure 2:**
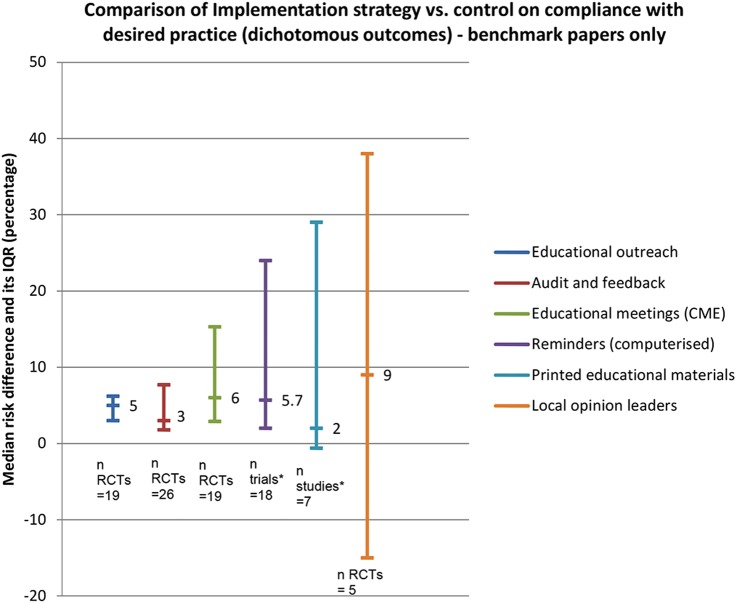
Graph illustrating median effects of single professional-level strategies alone versus no strategy or usual care. *Trials=inclusion of RCTs and quasi-experimental trial design; studies=inclusion of trials and non-trial design. CME, continuing medical education; RCT, randomised controlled trial.

The most frequently reported comparison was between the effectiveness of a single implementation strategy (eg, educational outreach or audit and feedback) and no strategy (table 1). The majority of these reviews reported dichotomous outcomes (or median improvement, often calculated as median RD) observed small to modest effects, ranging from 2% to 9%. Figure 2 illustrates the median effects and IQRs of single strategies targeted at professionals compared with no strategy or usual care, reported in the benchmark reviews. The lower IQR of educational outreach visits, audit and feedback, educational meetings and computerised reminders were all above zero (the line of no effect). Printed educational materials and local opinion leaders were the least effective single strategies. The IQRs of all strategies overlapped considerably, indicating that no single strategy appeared to be more effective than others.

Not all benchmark reviews provided results for continuous outcomes. The use of educational outreach visits was associated with the largest median change relative to no strategy (23%, IQR=12–39%), followed by educational meetings and workshops (10%, IQR=8–32%) and audit and feedback (1.3%, IQR=1.3–11%). In general, findings from non-benchmark reviews agreed with those from the benchmark reviews (table 1).

**Table 1:** Summary of the effects of single strategies and multifaceted strategies on adherence to desired practice

Strategy	Benchmark review Author, year (reference)	Outcome	Benchmark review results—single strategy alone vs no strategy	Benchmark review—details	Benchmark review —overall conclusion	Benchmark review—other comparisons	Benchmark reviews vs other (non-benchmark) reviews Overall results consistent with other relevant reviews*?
Professional-level strategies							
A&F	Ivers *et al*, 2012^34^	Compliance with desired practice	D*†: median absolute RD‡§=3% (IQR 1.8–7.7%)	26 RCTs (661 clusters/groups of health providers and 605 health professions); low-moderate risk of bias	Small (range: small to modest)	A&F with or without other strategies vs no strategy: **D**†: median RD‡§=4.3% (IQR 0.5–16.0%) (49 RCTs)	Yes^13^ ^16–33^
			**C**†: median percentage change relative to baseline control‡=1.3% (IQR 1.3–11%)	13 RCTs; low-moderate risk of bias	Not applicable	A&F with or without other strategies vs no strategy: median percentage change relative to baseline control‡=1.3% (IQR 1.3–28.9%) (21 RCTs)	
Physician reminder	Shojania *et al*, 2009^76^ Computer reminder (delivered at the point of care)	Improvement in process adherence	D*†: median RD¶=5.7% (IQR 2.0–24%)	18 RCT/quasi-randomised design	Modest (range: small to large)	Computer reminders with other strategies vs other strategies alone: **D**†: median RD¶=1.9% (IQR 0.0–6.2%; n trials not reported)	Yes^13^ ^19^ ^21^ ^22^ ^25^ ^28^ ^29^ ^37^ ^40^ ^49^ ^58^ ^60^ ^66–75^ ^77^
			**C**†: not reported		Not applicable	**C**†: not reported	
EOV	O'Brien *et al*, 2007^50^	Professional practice	D*†: median RD§¶=5% (IQR 3–6.2%)	19 RCT; low-moderate risk of bias	Small (range: small to modest)	EOV with or without other strategies vs no strategy: **D**†: median RD§¶=5.6% (IQR 3–9%; 28 RCTs)	Yes^13^ ^21^ ^22^ ^26–28^ ^32^ ^35^ ^37^ ^40^ ^45–49^
			**C**†: median adjusted change=23% (IQR 12–39%)	15 RCTs; low-moderate risk of bias	Not applicable	**C**†: median adjusted change=21% (IQR 11 to 41%; 17 RCTs)	
Educational meetings and workshops (including continuing medical education)	Forsetlund *et al*, 2009^12^	Compliance with desired practice	D*†: Median RD‡§=6% (IQR 2.9–15.3%)	19 RCTs; low-moderate risk of bias	Modest (range: small to moderate)	Educational meetings with or without other strategies vs no strategy: D†: median RD‡§=6% (IQR 1.8–15.9%) (30 RCTs)	Yes^19^ ^21^ ^22^ ^25^ ^31^ ^32^ ^35^ ^38^ ^41^ ^42^ ^47^ ^51–64^
			C†: median adjusted % change relative to the control group 10% (IQR 8–32%)	5 RCTs	Not applicable	C†: median adjusted % change relative to the control group 10% (IQR 9–24%) (8 RCTs)	
Local opinion leaders	Flodgren *et al*, 2011a^65^	Compliance with desired practice	D*†: median RD¶§=9% (IQR −15 to +38%)	5 RCT; high risk of bias	Modest and variable (range from negative, no effect, to small and large effects) Unclear due to inconsistent and limited evidence	Local opinion leaders alone or together with other strategies vs no intervention or other strategies alone D†: median RD¶§=12% (IQR 6–14.5%; 15 RCTs)	Mostly consistent: mixed effects^21^ ^22^ ^24^ ^32^ ^37^ ^40^
			C†: not reported			C†: not reported	
Printed educational materials (majority studies disseminated passively)	Giguère *et al*, 2012^44^	Professional practice	D*†: median RD¶=2% (IQR −0.6 to 29%)	7 studies; low quality	Small and variable (range: negative, no effect, to small and large effects)		Mixed but mostly consistent^13^ ^21–24^ ^26^ ^27^ ^32^ ^35–43^
			**C**†: SMD 13% (IQR 16–196%)	3 studies; low or very low quality			
Organisational-level strategies							
Revising professional roles	No benchmark review identified	28 61 69 78 83 84					
Facilitation	Baskerville *et al*, 2012^82^	Compliance with desired practice	SMD†=0.56 (95% CI 0.43 to 0.68; z=8.76; p<0.001; I^2^=20%) OR=2.76 (95% CI 2.18 to 3.43; non-significant heterogeneity, p=0.19)	20 RCTs and 3 CCTs (1398 participants); high quality	Effective (consistent)	Not applicable	Yes^78–81^
Context-level strategies							
Financial strategies	Scott *et al*, 2011^91^	Professional behaviours	All types of financial incentives, provided by primary care physicians Uncertain (no combined/overall effect size) Authors’ conclusion: different financial interventions had positive but modest and variable effects on a small number of outcome measures of quality of healthcare (7 studies)	7 studies	Variable High uncertainty	Not applicable	Yes. Some subsequent reviews presented positive results and some showed no effect or mixed results^20^ ^23^ ^78^ ^80^ ^85–90^
Regulatory strategies	None identified		Not applicable	Not applicable			Not applicable
Others							
Multifaceted strategies	No benchmark review identified	Multifaceted strategies likely to be more effective^27^ ^32^ ^36^ ^48^ ^51^ ^52^ ^60^ ^61^ ^71^ ^78^ ^105–113^ Multifaceted less or just as effective/unclear^12^ ^13^ ^17^ ^19^ ^20^ ^34^ ^50^ ^65^ ^76^ ^104^					
Tailored strategies to identified barriers	Baker *et al*, 2010^92^	Compliance with desired practice	Pooled adjusted OR†=1.54 (95% CI 1.16 to 2.01) from the Bayesian analysis Pooled OR=1.52 (95% CI 1.27 to 1.82) p<0.001 from the classical analysis	12 RCTs (2189 participants; moderate quality)	Not applicable	Not applicable	No other review identified

*Based on dichotomous data (intervention vs no intervention) from the benchmark review. Overall effect is described using the definition proposed by Grimshaw *et al*^13^ (see Methods).

†D, dichotomous; C, continuous; SMD, standardised mean difference.

‡Weighed according to the number of health professionals (number of practices, hospitals, communities) participating in the study.

§Adjusted for baseline differences in the outcome.

¶Unweighted or unclear weighting/adjustment.

A&F, Audit and feedback; CCT, controlled clinical trials; EOV, educational outreach visits; OR, odds ratio; RCT, randomised controlled trial; RD, risk difference.

###### Single strategy versus alternative single strategy

Only benchmark reviews of audit and feedback, local opinion leaders, printed educational materials and educational meetings reported direct head-to-head comparisons of these single strategies with alternative single strategy; this comparison was not commonly reported in primary studies. For example, only two trials with a moderate risk of bias compared educational meetings to other strategies, namely an educational outreach visit and a facilitated implementation of an office system to improve services. In both trials, educational meetings were associated with a decrease in compliance (adjusted RD of −1.4% and −8.0%), relative to the comparison strategies. Similarly, two trials compared opinion leaders alone to other strategies (standardised lectures and audit and feedback) and found a 14% absolute increase in adherence to desired practice for opinion leaders alone.^65^ No conclusions could be drawn from the limited evidence.

### Strategies directed at the organisation

#### Revising professional roles

We could not identify a benchmark review in this category. Six reviews examined the effects of revising professional roles, for example, having a nurse with a redefined role to offer support, such as undertaking preventive and follow-up tasks.^28^ ^61^ ^69^ ^78^ ^83^ ^84^ In general, these reviews demonstrated an improvement in process of care outcomes.

#### Practice facilitation

Five reviews^78–82^ examined the effects of practice facilitation, defined as having experienced facilitators, who can be internal or external to an organisation, to work with individual practices in order to facilitate and support a range of processes and activities, such as education, interactive consensus building and goal setting, quality improvement and problem solving. The benchmark review (total n=23 studies; 20 RCTs and 3 controlled clinical trials) reported an overall effect size of 0.56 (95% CI 0.43 to 0.68; p<0.001) which favoured practice facilitation (relative to controls) with non-significant heterogeneity and some indications of publication bias. It also found primary care practices are 2.76 (95% CI 2.18 to 3.43) times more likely to adopt evidence-based guidelines through practice facilitation.^82^ Similar significant effects were observed in other reviews.^79–81^ Practice facilitation improved adoption of guidelines in various clinical areas that focused on prevention, system-level improvements and outcomes associated with chronic disease management within practice settings.^79^

#### Changing organisational culture

One review assessed strategies to change organisational culture to improve professional practice.^93^ However, the authors were unable to draw conclusions about effective strategies for changing culture as no relevant primary studies fulfilled the methodological criteria for inclusion. There was a lack of reviews that summarised the evidence on organisational-level implementation strategies and little is known about what they might comprise.

#### Strategies directed at the wider context (eg, policy)

##### Financial strategies

Eleven reviews examined the effectiveness of financial strategies and the majority of these could not calculate an overall effect estimate due to heterogeneity, including the type of financial payment (eg, performance-based payment, capitation, fee-for-service), the size of payment, outcomes measured, targeted behaviour and the context/setting in which they were implemented. The benchmark review included seven studies and showed that financial strategies had positive but modest and variable effects on a small number of performance and quality of care outcomes.^91^ Other relevant reviews also reported mixed effectiveness. The majority of primary studies included in these reviews were conducted in the USA, and therefore may have limited applicability to other healthcare systems.

###### Effects of multifaceted strategies

Some reviews hypothesised that multifaceted implementation strategies could be more effective as more barriers could be addressed.^60^ However, the data suggested the effects of multifaceted strategies were variable and either no more effective or only slightly more effective in changing practice than single strategies.

All benchmark reviews assessed the effectiveness of their chosen strategy (or strategy of interest, eg, audit and feedback) plus additional strategies (more than one, eg, audit and feedback plus educational outreach visits), compared with no strategy; and the findings of this comparison group were largely similar to the findings of single strategies alone versus no strategy. Evidence from the remaining reviews (in the same category) also presented mixed results.^12^ ^13^ ^17^ ^19^ ^20^ ^27^ ^32^ ^34^ ^36^ ^48^ ^50^ ^51^ ^52^ ^60^ ^61^ ^65^ ^71^ ^76^ ^78^ ^104^ ^105–113^ Single strategies could be as effective as multifaceted strategies in improving practice particularly when baseline adherence to desired practice was low.

### Features of implementation strategies associated with success

Drawing on the literature included in this review of reviews, we identified features of implementation strategies that appeared to be associated with success. These are presented in table 2 and include features such as interactivity, tailoring and status of the individual delivering the strategy. Features that appeared to be relatively ineffective included didactic teaching format, low-intensity strategies and infrequent feedback.

**Table 2:** Features appeared to be associated with successful implementation

Strategy	Active features/characteristics	Inactive features/characteristics
Printed educational materials (PEM)	Tailoring Purpose (eg, increase or decrease in, modification of behaviour) Type of targeted behaviour Clinical area Format *Based on very limited evidence and box plots presented only	▸ Mode, Frequency, Duration of delivery are not associated with improvement in outcomes *Due to the lack of variability, not able to assess the importance of these characteristics to determine PEM effectiveness
Educational strategies	Mixed interactive and didactic formats High attendance at educational meetings Low complexity of the targeted behaviour Tailoring Relevance or identify needs with a facilitator Interaction/active participation Facilitate and (small) team based Training support Management support Clear goals Led by senior colleagues/superior Intensity and frequency Programmes directed at trainee physicians Focus on serious outcomes	Didactic sessions/lectures alone Seminar-based sessions High complexity of the targeted behaviour Minimal interaction/discussion Passive strategies (eg, mailed educational materials) Programmes directed at established physicians
Educational outreach visits	Most effective when the educators are known to and respected by the target group	No data reported
Audit and feedback (A&F)	Source—(p<0.001) supervisor/senior colleague Format—(p=0.02) feedback provided both verbally and written Measurable targets and action plan (p<0.001) Timing—concurrent feedback, presented close to the time of decision-making Active Tailoring Part of an overall strategy Low/ non-existent baseline	Effect size was not influenced by the number of implementation strategies in addition to A&F. A&F alone vs A&F in a multifaceted intervention: not significant; Dichotomous: estimated absolute difference in adjusted RD=3.3%, p=0.27)
Practice facilitation	Tailoring to the context and needs of the practice (SMD=0.62, 95% CI 0.48 to 0.75; p=0.05) Higher intensity of the intervention (average number of contacts by the average meeting time in hours; p=0.03) Smaller number of practices per facilitator (p=0.004)	No tailoring (SMD=0.37, 95% CI 0.16 to 0.58) Lower intensity of the intervention Larger number of practices per facilitator
Financial strategies	Larger size of payment Clear goal Low complexity of task Concurrent or intermittent payment Sustainability of new behaviour—incentives may only buy temporary priority Positive effect was greater for initially low performers (low baseline performance, more room for improvement) compared with already high performers Involvement of stakeholders in target selection and incentive programme development Context (national level gave more uniform results than fragmented programmes) Design choices (process indicators gave higher improvement than outcome measures) High awareness of the existence of an incentive programme Incentives based on financial rewards only showed more positive effects	Size of payment—small rewards may not motivate doctors to change their behaviour or practices High complexity of task End of year payment (infrequent performance feedback) Continuing adding additional funding or payment in the long term is not effective. Low awareness of the existence of an incentive programme Incentives based on a competitive approach (reward for high performers, as well as penalty for low performers)
Local opinion leaders	Multidisciplinary opinion leader teams	Single opinion leaders

A&F, audit and feedback; SMD, standardised mean difference.

### Evidence on economic evaluations

Overall there was a lack of economic evaluation data on the use of implementation strategies. Benchmark reviews mentioned that few primary studies reported costs or cost-effectiveness of the strategy.^50^

## Discussion

The purpose of this systematic review of reviews was to evaluate the effectiveness of strategies to improve implementation of complex interventions in primary care. We found that there has been a rapid increase in the number of primary studies and reviews examining the effectiveness of implementation strategies. Most of the included reviews evaluated the effects of individual professional-level implementation strategies and they may achieve small to modest improvement (range 2–9%) compared with no strategy. Of these professional-level strategies, educational outreach visits, educational meetings, and audit and feedback had the best evidence base; included a relatively large number of RCTs with low risk of bias. Passive dissemination strategies such as the distribution of educational materials appeared largely ineffective and the effect of local opinion leaders appeared variable.

There was a lack of evidence directly comparing the effectiveness of different strategies. These findings are largely consistent with those reported in a previous review of reviews on the effectiveness of professional-level strategies to promote the implementation of research findings.^94^ Although the median effects of most strategies were found to be small to modest, they might have much greater impact when applied at the population level, as 90% of care is delivered in primary care. Their effects may also be greater when applied in certain circumstances or settings. In addition, the follow-up period of the primary studies tended to be relatively short; therefore, long-term effects could not be determined.

There was limited review evidence on the effectiveness of organisational-level implementation strategies in primary care. There are some ongoing studies especially around promoting leadership and organisational culture, for instance, Curry *et al*^95^ have developed a theoretically informed intervention (multifaceted strategy approach) aimed at promoting organisational culture by encouraging organisational leadership which accelerates learning and improvement and integrated evidence-based practices into routine work of the organisation in 10 hospitals. Similarly Aarons *et al*^96^ conducted a randomised mixed-methods pilot study of a leadership and organisation development strategy for evidence-based mental health practice implementation. Further work is needed in this area, including identifying, describing and characterising potential organisational-level strategies and evaluating their effectiveness in any healthcare context. We identified even fewer reviews on strategies that addressed characteristics of the wider context level in primary care and most of these focused on financial arrangements and structures. None of the included reviews addressed regulatory strategies such as changes in medical liability laws, licensure standards and governance, or other wider context-level strategies, such as creating new funding for the use of a particular complex intervention or changes in policy.

Previous literature had suggested that multifaceted strategies could be more effective than single strategies,^94^ ^97^ ^98^ and their use was advocated in the 2008 Medical Research Council (MRC) complex intervention guidance as potentially useful approaches to implementation.^99^ However, we found that multifaceted implementation strategies were not necessarily more effective than single implementation strategies and that the effectiveness of multifaceted strategies did not increase incrementally with the number of components. Another recent systematic review of reviews examining whether multifaceted strategies are more effective than single strategies^100^ reported similar findings. There could be a number of possible reasons for this: (1) *ceiling effect*—both groups received co-strategies and any additional strategy would be unlikely to show further benefits; (2) *relevance*—strategies are often rarely justified theoretically,^8^ ^101^ that is, some strategies included are not necessary or relevant to the context; (3) *timing and delivery*—all the strategy components included in the primary studies might have been delivered at the same time, and possibly by spacing the components of multifaceted strategies at different times, may be more effective; (4) *active features* that support effective implementation were not included; and (5) strategies (in terms of combinations, timing/frequency, duration) and settings were too heterogeneous across primary studies to make it appropriate to combine them. In addition, multifaceted implementation strategies are likely to cost more than single implementation strategies.

Since the completion of this review, Powell *et al* compiled and published the Expert Recommendations for Implementing Change (ERIC) refined compilation of strategies for implementing change. This is a list of strategies for implementing clinical innovations in health and mental health based on sources such as published reviews and through expert consensus.^102^ We undertook a post hoc exercise and mapped the included reviews to the ERIC refined compilation of implementation strategies (see online supplementary file 7). We found that the evidence base for the majority of strategies included in this list was limited. This list is a valuable resource of discrete implementation strategies and more primary evaluation studies on the efficacy and effectiveness of these implementation strategies are required. Finally, we found very limited evidence on the cost-effectiveness of implementation strategies. Hoomans and Severens commented that despite the demand for undertaking economic evaluation in health services research, its use is not standard practice in assessing implementation strategies. They also found that studies on implementation strategies tend to assess only their effect on practice and health outcomes, and very few conducted economic evaluations.^103^

### Strengths and limitations

There are several strengths to this review of reviews. To the best of our knowledge, this is the most comprehensive review of the available literature on the effectiveness of single and multifaceted implementation strategies and is not restricted to any topic or health condition. It is therefore highly generalisable. The review was conducted using rigorous reviewing methods, including a comprehensive search strategy, double screening of all titles, abstracts and full-text articles, the use of a robust approach to selecting benchmark reviews, with findings elaborated with reference to other reviews. In addition, we were able to identify a tentative list of components of specific strategies that appeared to be associated with effective implementation.

There are also some limitations, including the possibility that not all relevant primary research studies were captured by included reviews, so some findings may be missed by concentrating on reviews. Moreover, by only focusing on reviews, there is an inevitable time lag, with recent studies less likely to be reported in reviews. Data extraction was conducted by a single reviewer. However, data extraction and synthesis of all benchmark papers plus two other randomly selected papers for each category were checked independently for accuracy by a second reviewer. There are a number of challenges to conducting this narrative synthesis: (1) the heterogeneous nature of the included primary studies and reviews (in terms of topic area, health conditions, type of analysis); (2) each review contained an enormous amount of information and we made a good attempt to focus on the results that best addressed our review question(s) by applying rigorous criteria and using a structured approach to synthesise the results.

#### Implications for clinical practice

Most implementation strategies targeted at changing practice at the professional level can achieve small to modest improvement. To facilitate successful implementation of complex interventions, the choice of strategies needs to be based on barriers relevant to the setting (context) in which the implementation occurs, in order to achieve maximum benefits. Furthermore, these barriers or implementation issues may change over time; they need to be reviewed periodically throughout the change process to ensure that the strategies used continue to be appropriate and relevant. In some circumstances, it may be more effective to use a single strategy and focus on one key problem of implementation instead of trying to tackle numerous problems using complex multifaceted strategies. When applying an implementation strategy, it is important to incorporate features shown to improve the likelihood of successful implementation.

#### Implications for research

This systematic review of reviews suggests that there is an increasing amount of primary and secondary research on the effectiveness of implementation strategies; however, they tended to focus on a small number of strategies with known evidence. Despite the large body of published literature, the evidence base on implementation strategies remains inconclusive. The evidence could not distinguish differences in effectiveness between various professional-level implementation strategies. Better designed (ie, development of strategies based on theoretical framework, tailored to relevant barriers) and described (ie, reporting of strategy components in accordance with reporting guidelines) studies are needed. Passive strategies alone are unlikely to be effective and in the authors’ opinion, no further studies of this kind are needed. Future research and systematic reviews should focus on why and how an implementation strategy (or combinations of strategies) works differently in different contexts and on more rigorous research testing a broad range of strategies that work at the organisational and wider contextual levels (What are they? How do they work? How effective and/or cost-effective are they?).

## Conclusion

The effects of professional-level implementation strategies were small to modest. Limited evidence was found in relation to the effectiveness of organisational-level and wider contextual-level implementation strategies. Our findings suggest multifaceted strategies may not always be more effective than a single strategy. Development and evaluation of implementation strategies should be informed by theoretical frameworks. There is no ‘one size fits all’ implementation strategy; they are likely to work best if tailored to local circumstances and takes account of broader policy context.
